# Joint effect of THBS2 and VCAN accelerating the poor prognosis of gastric cancer

**DOI:** 10.18632/aging.204520

**Published:** 2023-02-13

**Authors:** Long Wang, Li Feng, Linnan Liu, Jing Han, Xue Zhang, Dan Li, Jiayin Liu, Yudong Wang, Jing Zuo, Zhisong Fan

**Affiliations:** 1 Department of Oncology, The Fourth Hospital of Hebei Medical University, Shijiazhuang, Hebei 050000, PR China

**Keywords:** THBS2, VCAN, gastric cancer, gastritis, potential targets

## Abstract

Objective: Gastric cancer is the most common malignant tumor of the digestive system. The progression from gastritis to gastric cancer may be related to genetic factors, but the specific molecular mechanism remains unclear. Therefore, an in-depth study of the molecular mechanism of gastritis and gastric cancer is significant.

Methods: We downloaded two gene profiles, GSE2669 and GSE116312, from the Gene Expression Omnibus (GEO) database. This study aims to apply bioinformatics technology to mine differentially expressed genes (DEGs), DEGs annotation, protein-protein interaction (PPI) network creation, and hub gene identification and expression between gastric cancer patients and gastritis patients. Overall survival analysis of hub genes, analysis by comparative toxicogenomics database for hub genes in gastric cancer, THBS2 and VCAN protein expression by immunohistochemistry for gastric cancer and gastritis as well as design of the biological process (BP) neural network was implemented.

Results: The MSLN, SPP1, THBS2, SPARC, FN1, IGFBP7, VCAN were up-regulated in gastric carcinoma samples, while FGA was down-regulated. The protein expression of THBS2 and VCAN in gastric cancer was significantly higher than that in gastritis. VCAN protein expression was positively associated with tumor invasion (*P* = 0.011) and HER2 overexpression (*P* = 0.031). Strong correlation among THBS2, VCAN, and gastric cancer based on the BP neural network.

Conclusion: THBS2 and VCAN may be potential targets for improving gastric cancer patients' diagnosis and clinical efficacy.

## INTRODUCTION

Gastric cancer is a malignant tumor originating from gastric mucosa, mainly due to gastric mucosal epithelial lesions. It is a public health problem endangering China and even the world. In recent years, the new cases of gastric cancer have been increasing, and its mortality rate ranks third in the world [1]. The occurrence of gastric cancer might be closely associated with the adverse environment, lifestyle, and changes in diet structure in the population. The symptoms of early gastric cancer are not obvious, and some patients may have dyspepsia [2]. The symptoms of advanced gastric cancer may include upper abdominal pain, postprandial aggravation, poor appetite, anorexia, fatigue, and weight loss. Because gastric cancer patients have no obvious clinical symptoms or mild symptoms, patients easily ignore it. Once obvious clinical symptoms appear and have been advanced, the prognosis is significantly reduced. Therefore, early diagnosis and treatment of gastric cancer are critical [3]. Gastric cancer’s development was slow, mainly experiencing different processes of gastritis - atrophy - intestinal metaplasia - atypical hyperplasia. One of the characteristics of precancerous lesions of gastric cancer is chronic atrophic gastritis accompanied by atypical metaplasia of the intestinal epithelium. Atrophy, intestinal intraepithelial neoplasia of gastric mucosa glands might be closely associated with gastric cancer. The progression from gastritis to gastric cancer might be closely associated with genetic factors, but the specific molecular mechanism remains unclear. Therefore, the potential mechanism of gastritis and gastric cancer is fundamental.

THBS2 is a member of the Ca^2+^ binding glycoprotein family of stromal cells. It has been found that THBS2 interacts with a variety of cell receptors, growth factors, and extracellular matrix (ECM) proteins, leading to its functions in cell adhesion, proliferation, and apoptosis [4, 5]. VCAN, a chondroitin sulfate proteoglycan, is a member of the proteoglycan family and is involved in forming and maintaining tissue shape. VCAN protein is abundant in the temporary matrix generated in the early stages of development and illness through interacting with numerous protein molecules. It might be closely associated with cell classification, proliferation, adhesion, and migration [6–8]. However, its relationship with gastritis and gastric cancer remains unclear.

Bioinformatics is a new discipline that collects and analyzes genetic data [9, 10]. Furthermore, it takes genomic DNA sequence information analysis as its source, which has been widely used in the mining and exploration of tumor-related hub genes [11, 12].

Therefore, the study aims to use bioinformatics to mine the hub genes between gastric cancer patients and gastritis patients and perform enrichment and survival analysis. Furthermore, the protein expression difference of THBS2 and VCAN were detected in human gastritis and gastric cancer, and the correlation between THBS2 and VCAN and gastric cancer was speculated.

## MATERIALS AND METHODS

### Dataset

GSE2669 and GSE116312 were downloaded from the Gene Expression Omnibus (GEO) database, containing gene expression data submitted by research institutions, including gene chip and high-throughput sequencing data. The GSE2669 includes 66 gastric carcinoma tissues and 26 gastritis tissues. Furthermore, GSE116312 includes 3 gastric carcinoma tissues and 10 gastritis tissues.

### DEGs identification

GEO2R is software for differential analysis of expression spectrum chips based on the GEO database. This study performed differential analysis for the GSE2669 and GSE116312 by GEO2R. The log (Fold Change) >1 or <-1 and *P* < 0.05 were defined as cut-off criteria.

### DEGs annotation

The enrichment analysis was performed by the Metascape, Database for Annotation, Visualization and Integrated Discovery (DAVID), and GSEA (Gene Set Enrichment Analysis). Metascape integrates more than forty bio information databases, which could make enrichment analysis. The DAVID integrates plenty of biological data to provide comprehensive functional annotation. The GSEA uses a predefined gene set to order genes in two types of samples and then check the enrichment score of the genes.

### Interaction among the proteins coded by DEGs

The Search Tool for the Retrieval of Interacting Genes (STRING) records possible and plausible protein interactions. Protein-protein interaction (PPI) network was constructed via the STRING tool, and Cytoscape (version 3.8.0) was performed to visualize the network.

### Analysis for the hub genes

Molecular Complex Detection tool (MCODE) (version 1.6.1) is based on the relationship between edge and node in the vast network and could find out the critical sub-network and gene, convenient for downstream analysis. The criteria MCODE scores >5 was the cut-off criterion. Moreover, the hub genes of gastric cancer were identified via MCODE.

### Effect of the hub genes expression on the survival time of gastric cancer patients

Kaplan Meier-plotter (http://kmplot.com/analysis/index.php?p=service) is an online tool for survival analysis. Currently, this tool includes numerous high-throughput sequencing data involving mRNA and miRNA. After screening the biomarkers in relevant research data, the tool is then used to analyze the survival outcomes of patients with differential gene expression. Therefore, the effect of the hub genes expression on the survival time of gastric cancer patients was verified by this tool.

### Analysis by comparative toxicogenomics database (CTD) for hub genes in the gastric cancer

The CTD provides information on the complex interactions between chemical exposure, genes, proteins, phenotypes, and disease. These data are integrated with functions to help develop hypotheses about the underlying mechanisms of diseases affected by the environment.

### Human samples

Seventy-eight paraffin-embedded gastric cancer samples were collected from the Fourth Hospital of Hebei Medical University. These patients, including 59 males and 19 females, underwent surgery from May 2020 to October 2020. None of these patients had undergone preoperative radiation or chemotherapy before surgery. All tissues were confirmed as gastric adenocarcinoma by pathological diagnosis. We used the 8th edition of the American Joint Committee on Cancer stage system for TNM stage. In addition, 22 endoscopic biopsy tissues of gastritis patients including 12 males and 10 females were collected from March 2022 to April 2022 in the Second Hospital of Hebei Medical University. The biopsy tissues were confirmed as gastritis by pathological diagnosis.

### Immunohistochemistry

Immunohistochemistry (IHC) was used to detect the protein expression of VCAN and THBS2. The high temperature and high pressure antigen retrieval method was used for antigen retrieval. The sections were separately added with Anti-THBS2 rabbit polyclonal antibody (Sangon Biotech Co., Ltd, Shanghai, China) or Anti-VCAN rabbit polyclonal antibody (Sangon Biotech Co., Ltd, Shanghai, China) and incubated in 2 hours. Then these sections were added with Goat anti-Rabbit IgG (Sangon Biotech Co., Ltd, Shanghai, China) and incubated in 20 min. Then Diaminobenzidine tetrahydrochloride (DAB) staining was used for staining. The immunohistochemical results were interpreted by two pathologists. THBS2 and VCAN protein expressions were evaluated by intensity of staining and percentage of stained tumor cells. Intensity was given scores 0–3 (0, no; 1, weak; 2, moderate; 3, strong) and the percentage of stained tumor cells in all tumor cells was given scores 0–4 (0:<5%, 1:5–25%, 2:>25–50%, 3:>50–75%, 4:>75%). The semiquantitative immunoreactivity scoring system (IRS) was used to score, that is the percentage fraction of stained tumor cells multiplied by the staining intensity fraction. The positive expression was defined as the score >3 and the negative expression was defined as the score ≤3.

### Design of BP neural network

BP neural network could use an error backpropagation algorithm to solve the parameter tuning problem in a neural network. Due to the limitation of data, part of the input feature vectors in the disease risk determination model was reduced. Finally, the three parameters of THBS2 and VCAN, disease of gastric cancer, and gastritis, were defined as input data. Corresponding to the three characteristics data entered is the individual’s diagnostic result. In the health status item, 1 represents that the individual is gastric cancer, and 0 represents that the individual is gastritis. MATLAB was used to construct the BP neural network.

### Statistics

The study used percentage, mean and standard deviation to describe the data. *T* test, χ^2^ tests or Fisher’s exact probability tests were used to analyze differences between groups. SPSS 25.0 software was used to make statistical analysis.

### Availability of data and materials

The datasets used and/or analyzed during the current study are available from the corresponding author on reasonable request.

## RESULTS

### Difference between gastric cancer and gastritis

**Figure 1:**
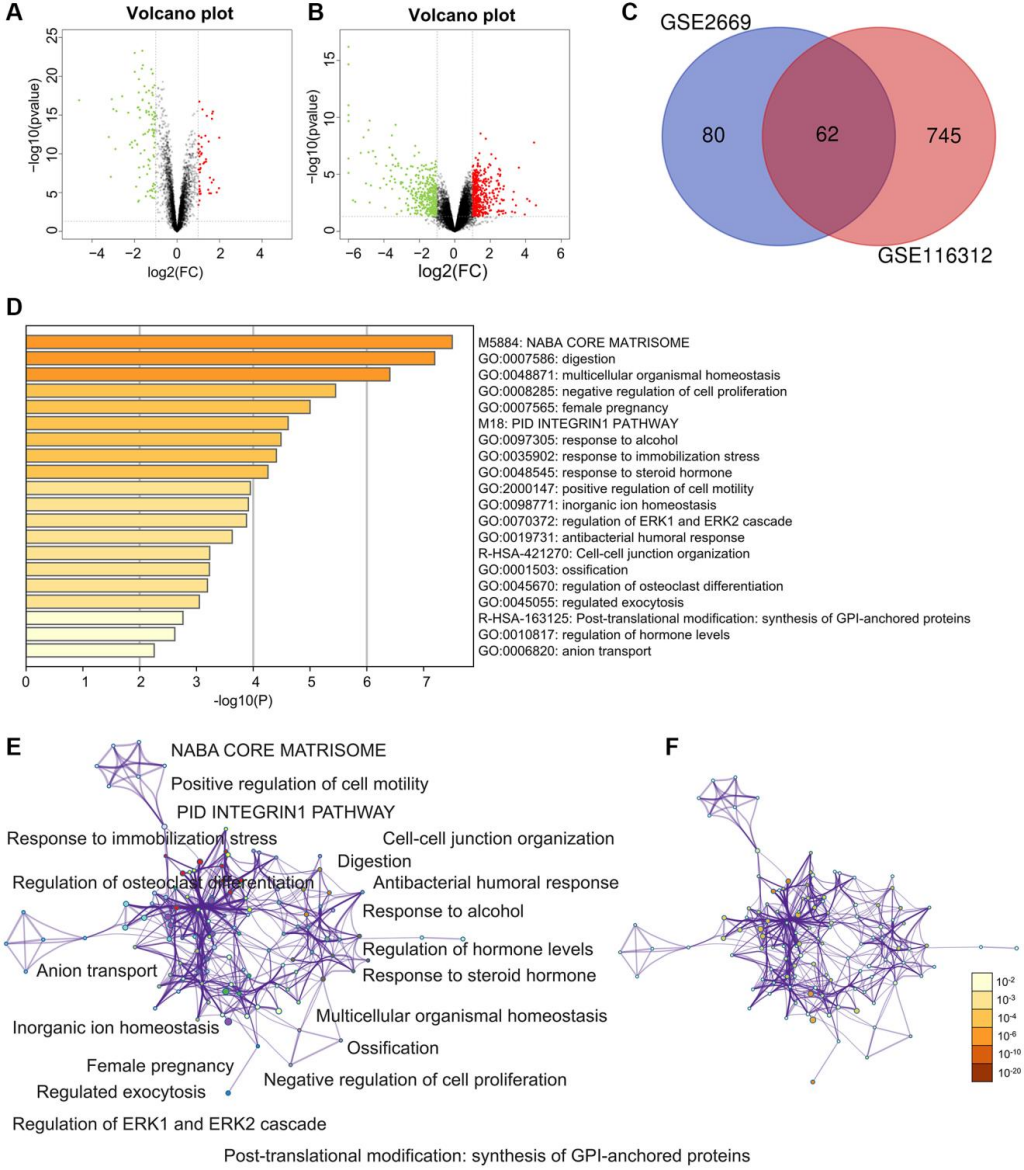
**Differently expressed genes (DEGs) and enrichment analysis for the DEGs by Metascape.** (**A**) The DEGs in the GSE2669. (**B**) The DEGs in the GSE116312. (**C**) The common DEGs between GSE2669 and GSE116312. (**D**) Heatmap of enriched terms across input differently expressed gene lists, colored by *p*-values, via the Metascape. (**E**) Network of enriched terms colored by cluster identity, where nodes that share the same cluster identity are typically close. (**F**) Network of enriched terms colored by *p*-value, where terms containing more genes tend to have a more significant *p*-value.

There were plenty of DEGs between gastric cancer and gastritis in the GSE2669 (Figure 1A) and GSE116312 (Figure 1B). A total of 62 DEGs appeared simultaneously in the two datasets (Figure 1C).

### Enrichment analysis for DEGs

**Figure 2:**
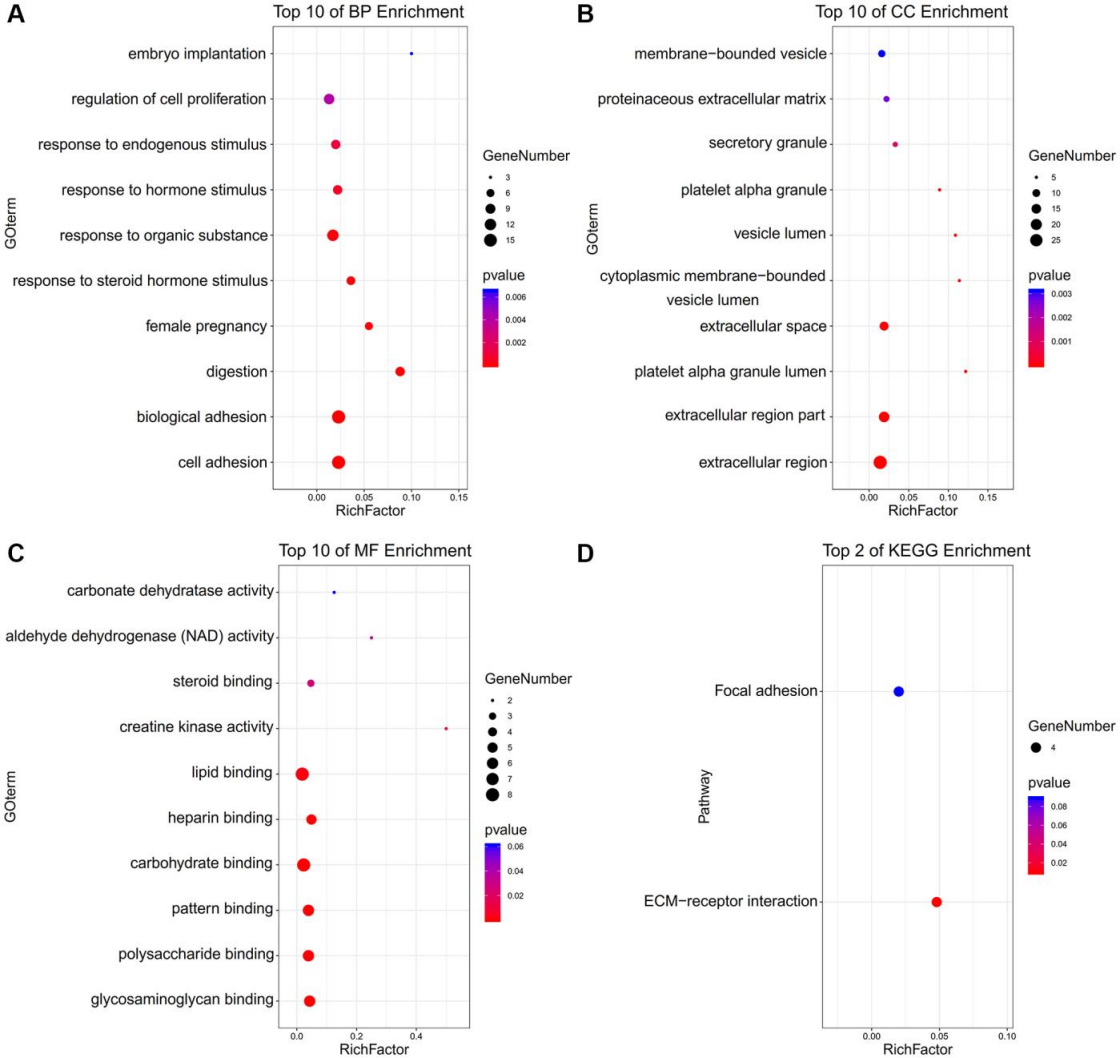
**The functional annotation for the DEGs based on the DAVID.** (**A**) BP, (**B**) CC, (**C**) MF, and (**D**) KEGG.

The enriched terms in Metascape were described in Figure 1D–1F. In an aspect of biological process (BP), DEGs were enriched in embryo implantation, regulation of cell proliferation, female pregnancy, digestion, biological adhesion, cell adhesion (Figure 2A). In an aspect of cellular component (CC), DEGs were enriched in the membrane-bounded vesicle, proteinaceous extracellular matrix, vesicle lumen, extracellular space, extracellular region part, extracellular region (Figure 2B). In the aspect of molecular function (MF), DEGs were enriched in carbonate dehydratase, aldehyde dehydrogenase (NAD), steroid, creatine kinase activity, lipid, heparin, carbohydrate, pattern, polysaccharide, glycosaminoglycan (Figure 2C). Focal adhesion and ECM-receptor interaction were the main enriched terms in the KEGG pathway (Figure 2D).

**Figure 3:**
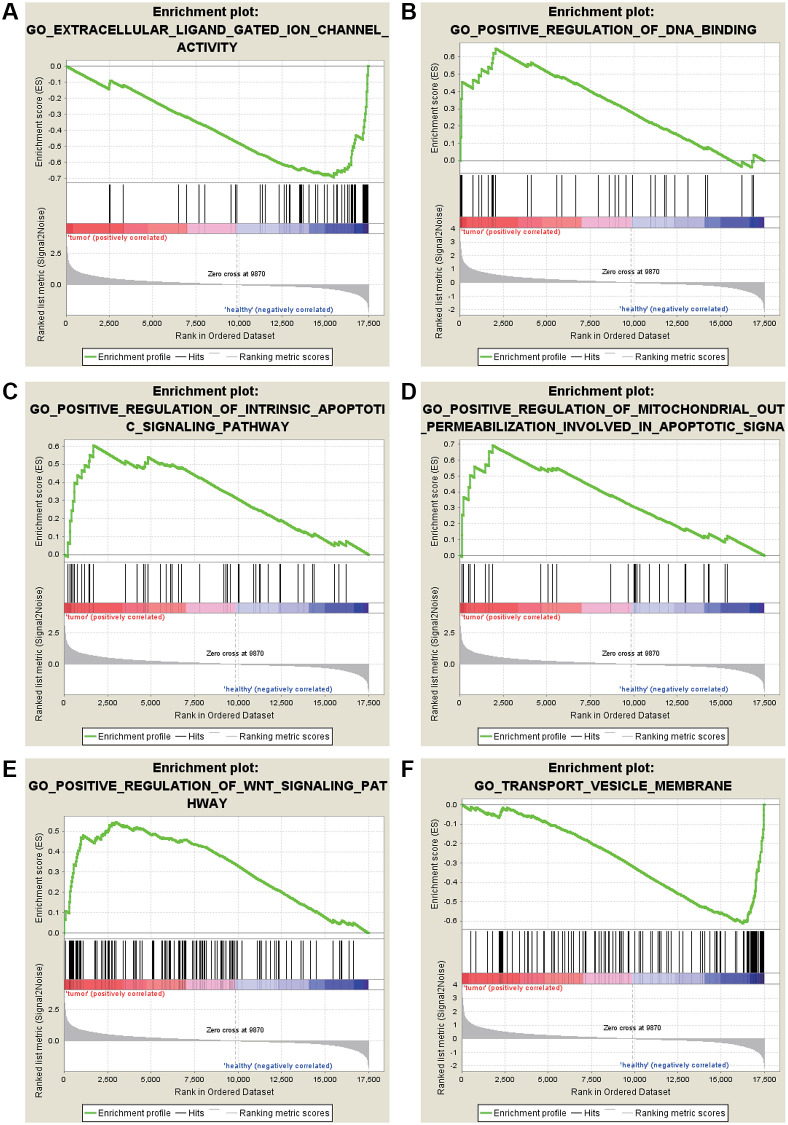
**GO enrichment analysis for the DEGs by GSEA.** (**A**) Extracellular ligand gated ion channel activity. (**B**) Positive regulation of DNA binding. (**C**) Positive regulation of intrinsic apoptotic signaling pathway. (**D**) Positive regulation of mitochondrial out permeabilization involved in apoptotic signaling pathway. (**E**) Positive regulation of WNT signaling pathway. (**F**) Transport vesicle membrane.

The most significant GO enrichments for gene sets of gastric cancer in the significant order were “EXTRACELLULAR LIGAND GATED ION CHANNEL ACTIVITY” “TRANSPORT VESICLE MEMBRANE” (Figure 3).

**Figure 4:**
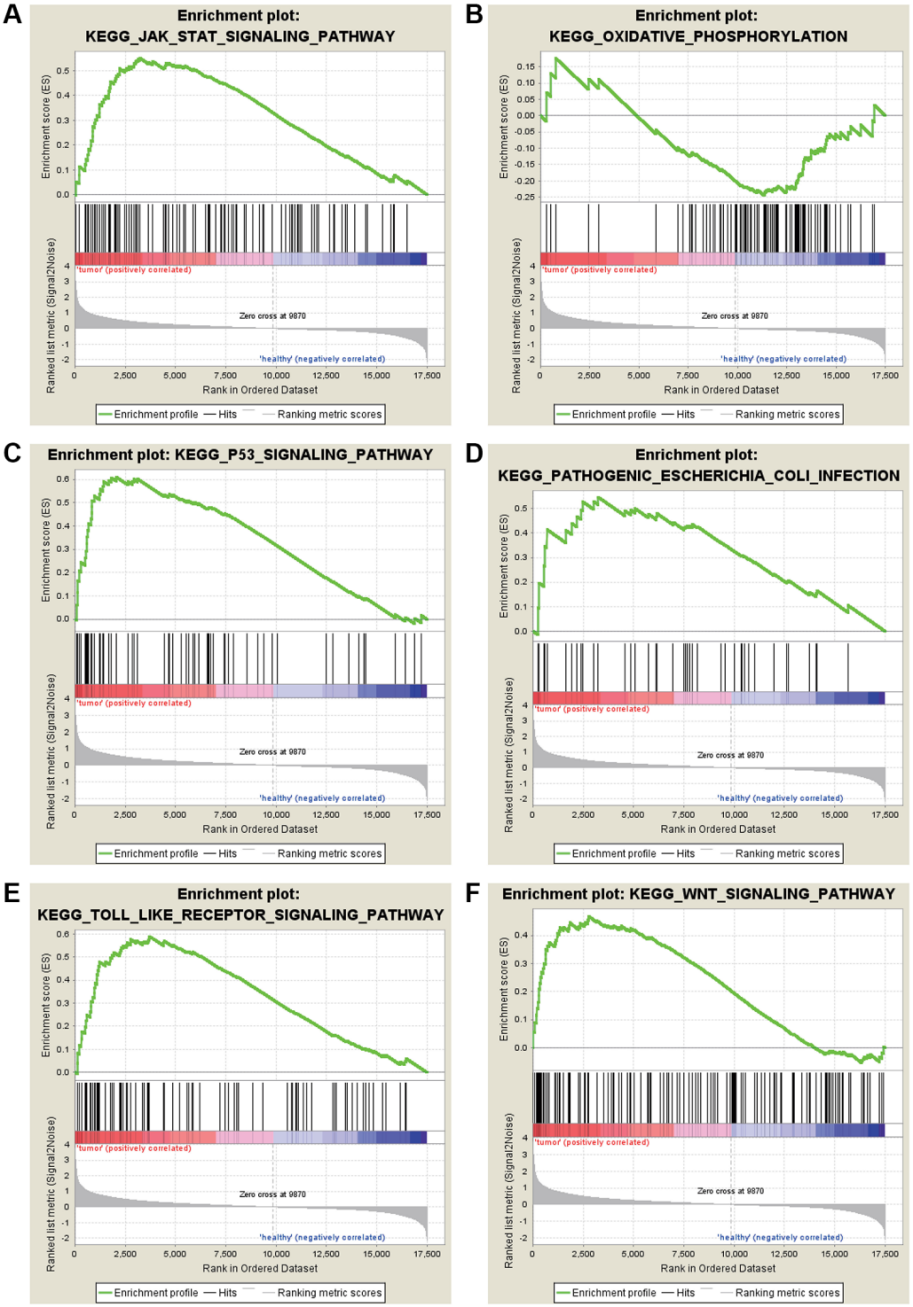
**KEGG enrichment analysis for the DEGs by GSEA.** (**A**) JAK-STAT signaling pathway. (**B**) Oxidative phosphorylation. (**C**) P53 signaling pathway. (**D**) Pathogenic *Escherichia coli* infection. (**E**) TOLL like receptor signaling pathway. (**F**) WNT signaling pathway.

The most significant KEGG enrichment for gene sets of gastric cancer in the significant order was “JAK-STAT SIGNALING PATHWAY,” “OXIDATIVE PHOSPHORYLATION,” and “P53” (Figure 4).

### PPI and hub genes

**Figure 5:**
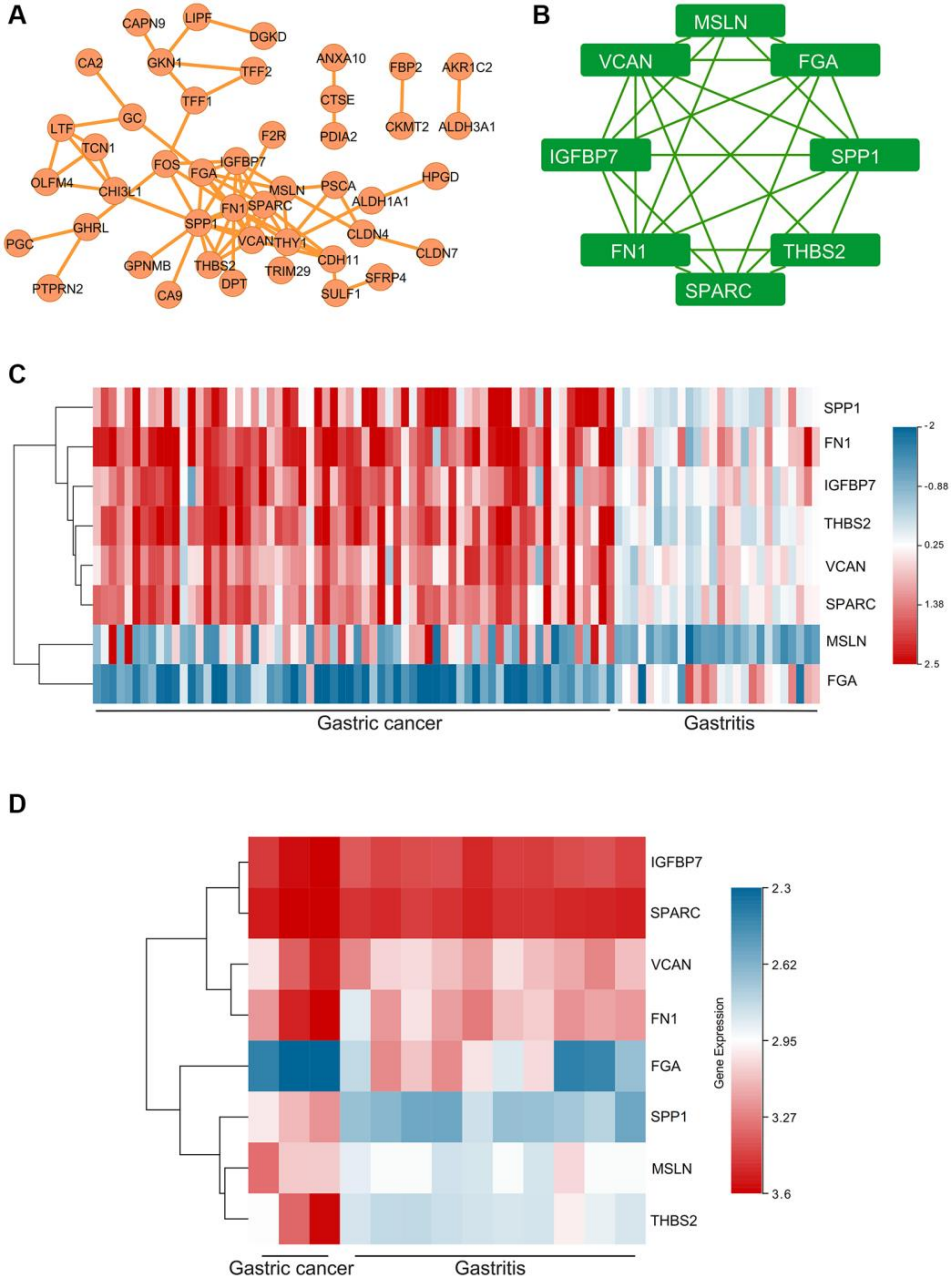
**The PPI network and hub genes, expression analysis for the hub genes.** (**A**) PPI network. (**B**) The key module of MCODE analysis. (**C**) The heat map showed the expressions of the hub genes in GSE2669. (**D**) The heat map showed the expressions of the hub genes in GSE116312.

PPI network showed the closed interactions among DEGs (Figure 5A). The key module of MCODE analysis was shown, which included MSLN, FGA, SPP1, THBS2, SPARC, FN1, IGFBP7, VCAN (Figure 5B). Expressions of hub genes in GSE2669 (Figure 5C) and GSE116312 (Figure 5D) were presented via the heat maps. Moreover, the MSLN, SPP1, THBS2, SPARC, FN1, IGFBP7, VCAN were up-regulated in gastric carcinoma samples, while FGA was down-regulated.

### Overall survival of gastric cancer patients

**Figure 6:**
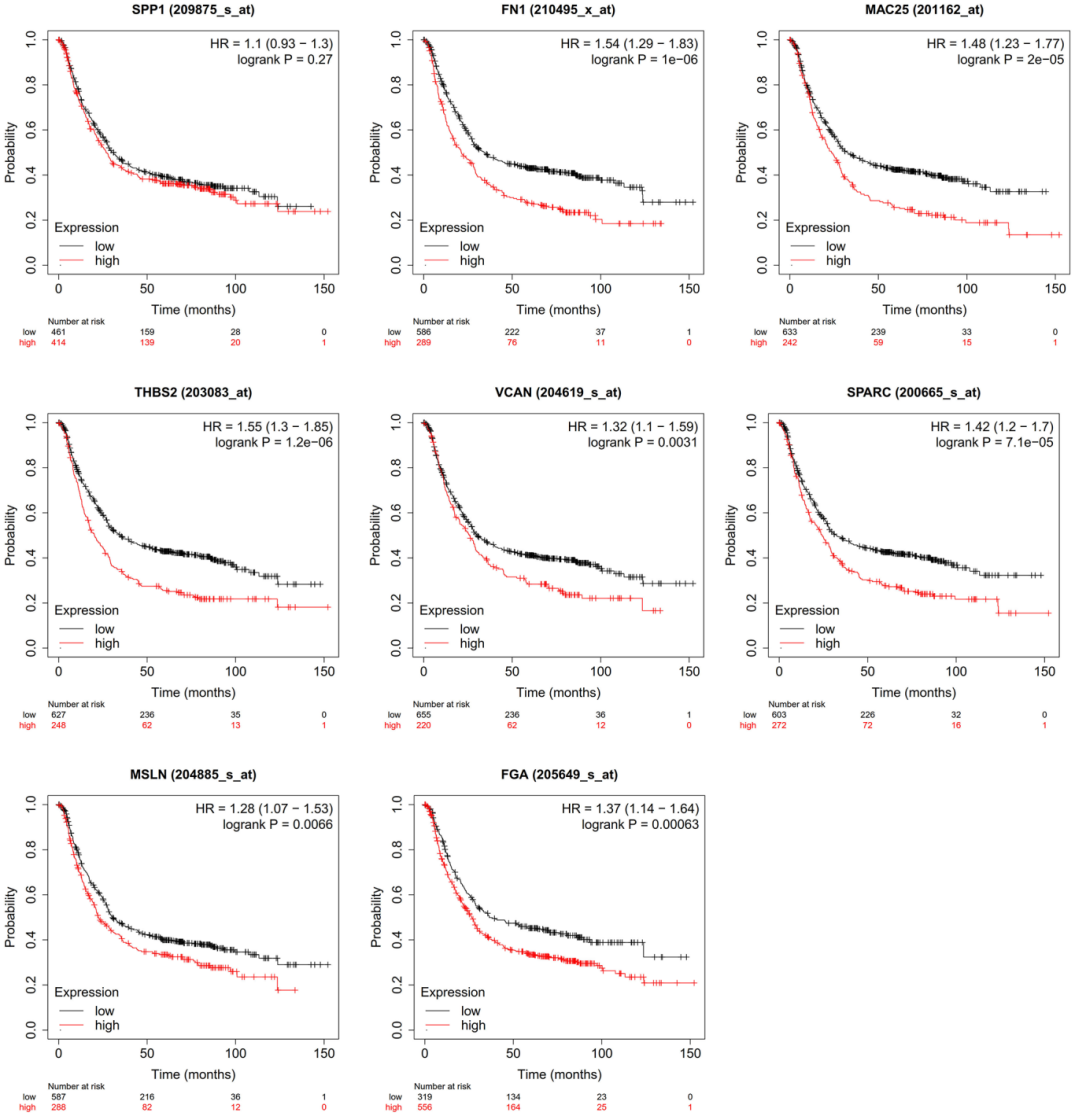
Effect of hub genes on the overall survival of gastric cancer.

There was no relationship between gastric cancer and SPP1 (*P* > 0.05). Gastric cancer patients with high expression levels of FN1 (HR = 1.54, *P* < 0.05), MAC25 (HR = 1.48, *P* < 0.05), THBS2 (HR = 1.55, *P* < 0.05), VCAN (HR = 1.32, *P* = 0.0031), SPARC (HR = 1.42, *P* < 0.05), MSLN (HR = 1.28, *P* = 0.0066), and FGA (HR = 1.37, *P* < 0.05) had shorter survival time (Figure 6).

### Identification of inference score of hub genes in the gastric cancer

**Figure 7:**
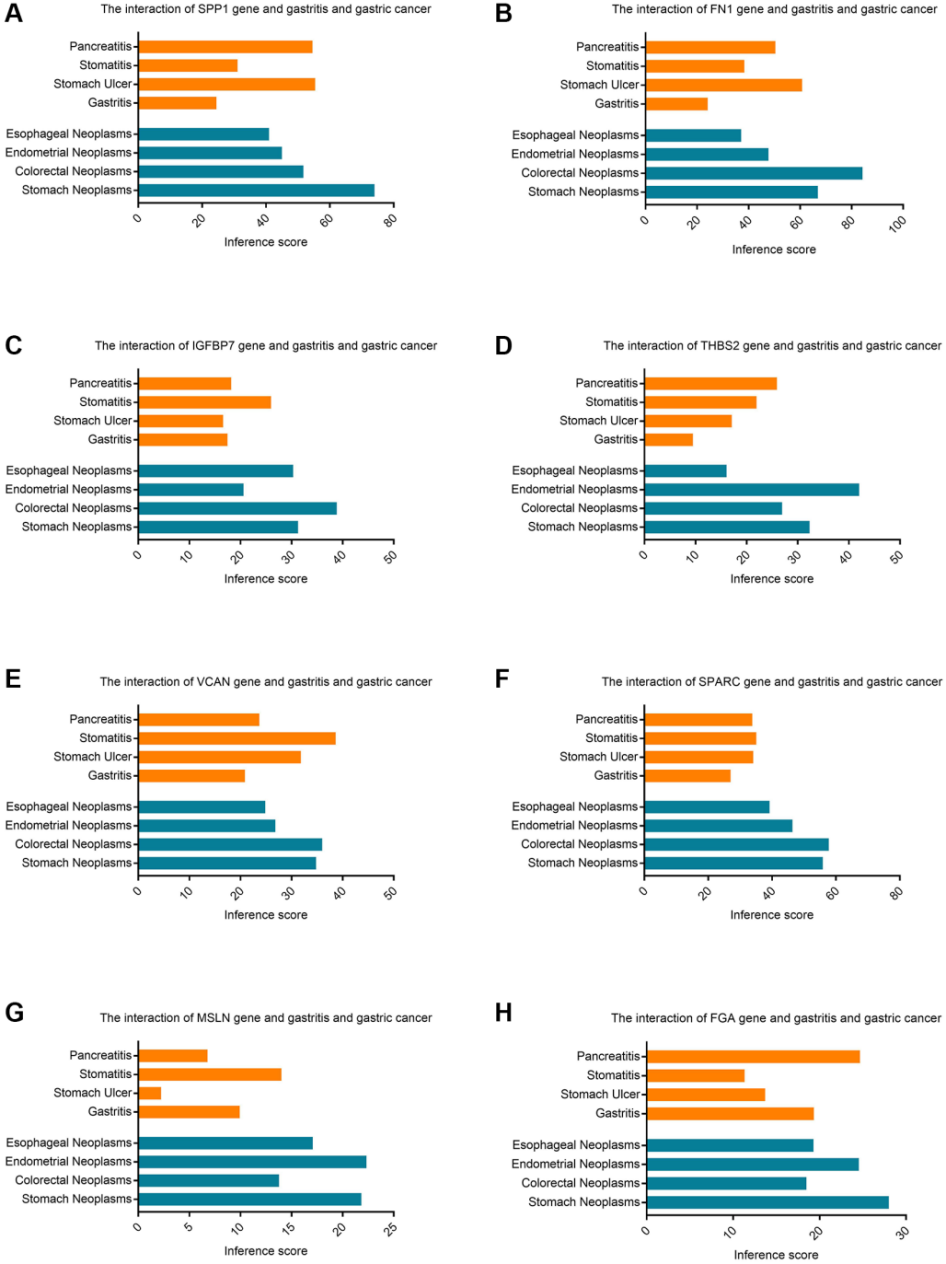
**The CTD database identifies the inference score of hub genes in gastric cancer.** (**A**) The interaction of SPP1 gene and gastritis and gastric cancer. (**B**) The interaction of FN1 gene and gastritis and gastric cancer. (**C**) The interaction of IGFBP7 gene and gastritis and gastric cancer. (**D**) The interaction of THBS2 gene and gastritis and gastric cancer. (**E**) The interaction of VCAN gene and gastritis and gastric cancer. (**F**) The interaction of SPARC gene and gastritis and gastric cancer. (**G**) The interaction of MSLN gene and gastritis and gastric cancer. (**H**) The interaction of FGA gene and gastritis and gastric cancer.

The CTD manifested that significant hub genes targeted gastric cancer, and the data were shown in Figure 7. There existed substantial value of MSLN, FGA, SPP1, THBS2, SPARC, FN1, IGFBP7, VCAN on the development and occurrence of gastric cancer and gastritis.

### The protein expression of THBS2 and VCAN in the gastric cancer and gastritis

**Figure 8:**
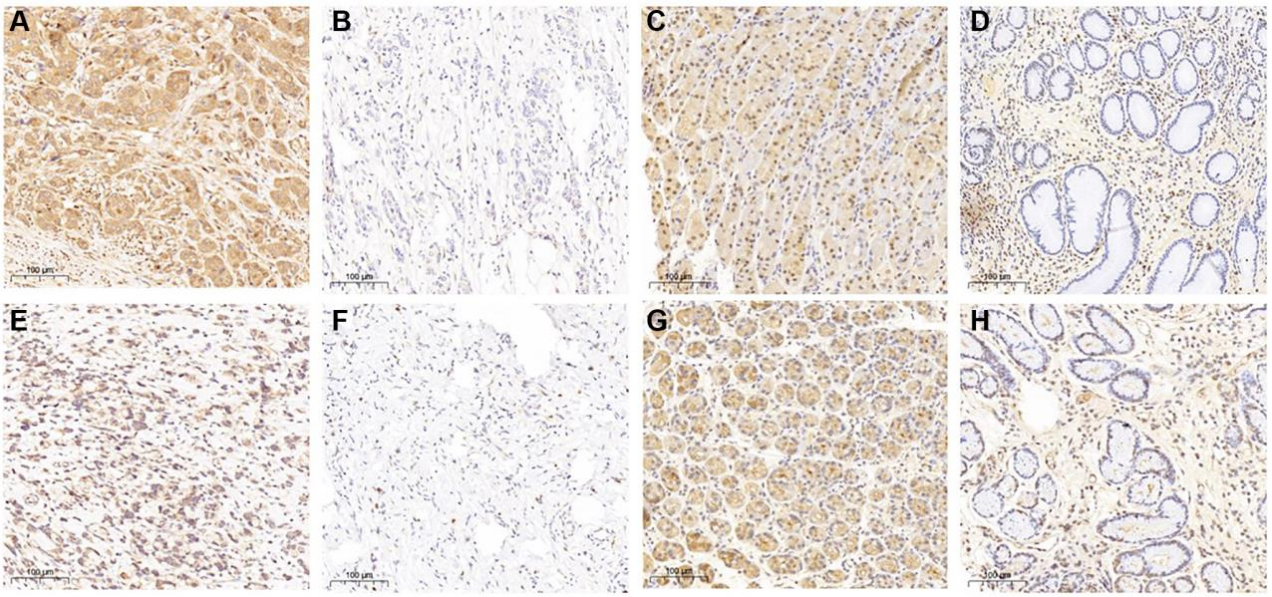
**THBS2 and VCAN expression in gastric cancer and gastritis by immunohistochemistry.** THBS2 protein was mainly expressed at the cytoplasm. The expression of THBS2 protein in gastric cancer was significantly higher than that in gastritis (*P* = 0.003). (**A**) Positive expression of THBS2 protein in gastric cancer; (**B**) Negative expression of THBS2 protein in gastric cancer; (**C**) Positive expression of THBS2 protein in gastritis; (**D**) Negative expression of THBS2 protein in gastritis. VCAN protein expressed mainly at the cytoplasm and cell membrane. The expression of VCAN protein in gastric cancer was significantly higher than that in gastritis (*P* < 0.001). (**E**) Positive expression of VCAN protein in gastric cancer; (**F**) Negative expression of VCAN protein in gastric cancer; (**G**) Positive expression of VCAN protein in gastritis; (**H**) Negative expression of VCAN protein in gastritis.

IHC was used to explore the protein expression of THBS2 and VCAN in the gastric cancer and gastritis (Figure 8). THBS2 protein was positively expressed in 87.18% (68/78) gastric cancer tissues and 59.09% (13/22) of gastritis tissues. The expression of THBS2 protein in gastric cancer was significantly higher than that in gastritis (*P* = 0.003). While, the expression of THBS2 protein in gastric cancer did not have correlation with TNM stage, the depth of tumor invasion depth, lymph nodes metastases, HER2 expression or the Lauren classification (Table 1). Positive expression of VCAN protein was found in 91.03% (71/78) gastric cancer tissues and 4.55% (1/22) gastritis tissues. The expression of VCAN protein in gastric cancer was significantly higher than that in gastritis (*P* < 0.001). VCAN protein expression was significantly positively associated with tumor invasion depth (*P* = 0.011) and HER2 protein expression (*P* = 0.031). There was no association between the expression of VCAN protein and TNM stage, lymph nodes metastases or the Lauren classification (Table 1). In addition, there was no correlation between VCAN and THBS2 protein in gastric cancer (Table 1).

**Table 1:** Association between the protein expression of THBS2 and VCAN and clinicopathologic parameters in 78 patients with gastric cancer.

**Clinicopathologic features**	**THBS2 protein**		***P***	**VCAN protein**		***P***
	**Positive (*n* = 71)**	**Negative (*n* = 7)**		**Positive (*n* = 71)**	**Negative (*n* = 7)**	
**TNM stage**						
II	12	2	0.856	11	3	0.072
III	56	8		60	4	
**Tumor depth**						
T1	1	0	0.445	0	1	0.011
T2	8	1		9	0	
T3	1	1		2	0	
T4	58	8		60	6	
**Lymph nodes metastases**						
N0	7	1	0.998	6	2	0.299
N1	14	2		14	2	
N2	22	3		24	1	
N3	25	4		27	2	
**Age**						
≤60	33	8	0.063	36	5	0.295
>60	35	2		35	2	
**Gender**						
Male	55	6	0.135	57	4	0.157
Female	13	4		14	3	
**HER2 expression**						
Positive	6	0	0.324	4	2	0.031
Negative	61	10		66	5	
**Lauren typing**						
Intestinal-type	7	2	0.125	9	0	0.401
Diffuse-type	22	6		26	2	
Mixed-type	35	2	32	5
**VCAN protein**						
Positive	61	10	0.288			
Negative	7	0				

### Strong correlation among the THBS2, VCAN, and gastric cancer based on the BP neural network

**Figure 9:**
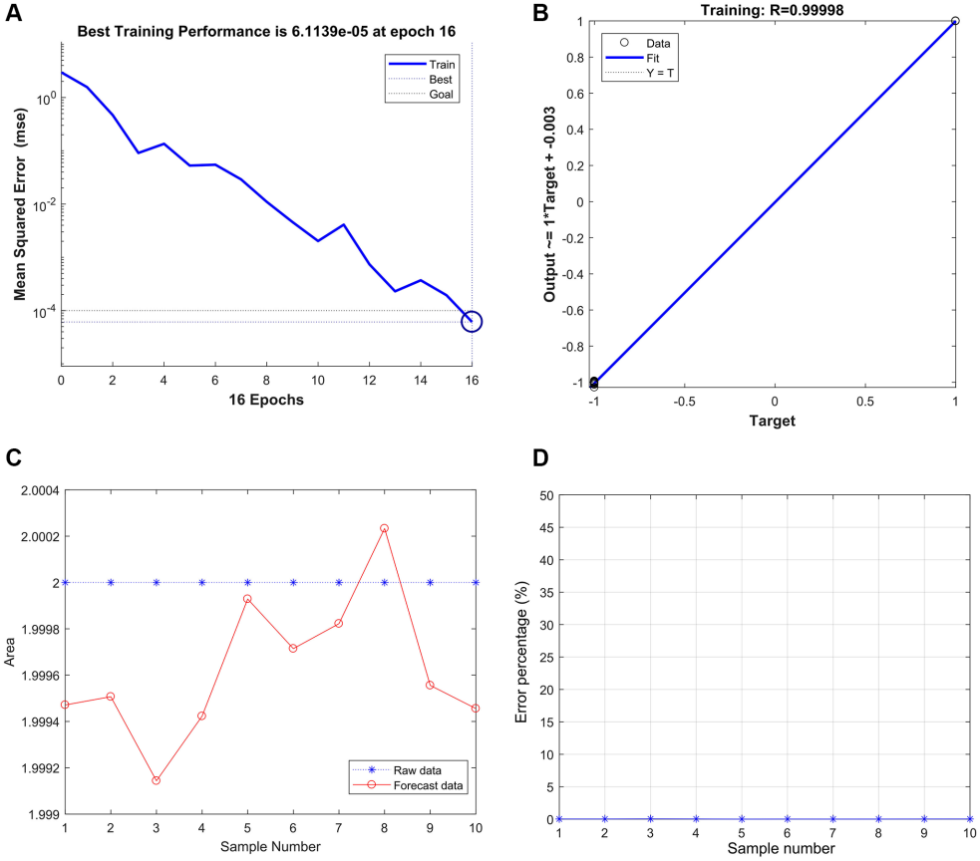
**Strong correlation between THBS2, VCAN, and gastric cancer based on the BP neural network.** (**A**) Best Training Performance is 6.1139e-05. (**B**) The relativity was 0.99998. (**C** and **D**) The model verified the result, and there were only no significant differences between the predicted and actual values.

The best training performance is 6.1139e-05 at epoch 16 (Figure 9A), and the relativity was 0.99998 (Figure 9B). Then, the model verified the result, and there were only no significant differences between the predicted and actual values (Figure 9C and 9D).

## DISCUSSION

Gastric cancer’s pathogenesis is mainly related to genetic material changes, helicobacter pylori, Epstein-Barr virus (EBV) infection, and epigenetic changes [13–15]. The prognosis is relatively poor. More than 80% of gastric cancers are diagnosed at an advanced stage in China. Patients may miss the chance of radical resection or have a high risk of postoperative metastasis and recurrence [16]. Although the combined application of surgery, radiotherapy, chemotherapy, and targeted therapy has prolonged the survival time, its pathogenesis was unclear, so the prognosis of gastric cancer patients is still not ideal [17, 18]. Our study found that compared with gastritis patients, THBS2 and VCAN were up-regulated in gastric cancer patients, and these individuals had a shorter survival time.

THBS2, encodes an extracellular matrix glycoprotein from the THBS family with a molecular weight of 20 kD secreted by fibroblasts and smooth muscle cells, located on chromosome 6q27 [19]. It binds to several cell receptors, growth factors, and intercellular matrix proteins to regulate cell adhesion, motility, wound healing, and embryo development. It has numerous sites that bind to thrombin, fibrinogen, fibrinolytic protein, heparin, and other substances. Meng et al. [20] suggested that the THBS2 reduced vascular distribution, progression and metastasis. Sun et al. [21] found that gastric cancer patients with higher THBS2 levels had a better prognosis, while decreased THBS2 expression was associated with poor gastric cancer histological grade and poor prognosis. While, several studies have found that the higher THBS2 was correlated with the shorter overall survival [5, 22, 23]. In our study, THBS2 protein has higher expression in gastric cancer than in gastritis and was adverse prognostic factor for gastric cancer patients. So we speculated that THBS2 might inflammatory gastric mucosa lesions and change the local microenvironment of gastric mucosa during inflammation. THBS2 may also affect the differentiation and infiltration of tumors by inhibiting the formation of tumor blood vessels.

Versican (VCAN), located on chromosome 5q12-14, encodes four proteoglycan isoforms, which can interact with other proteins through highly negatively charged chondroitin/dermal sulfate side chains and G1 and G3 domains [24]. Abnormal expression of VCAN protein is closely related to the poor prognosis of a variety of tumors. Studies *in vitro* and *in vivo* have shown that VCAN regulates various cellular processes, including tumor phenotypes and properties, development of drug resistance, and tumor-stromal angiogenesis [25, 26]. Previous relevant studies have shown that VCAN is highly expressed in various malignant tumors, such as kidney cancer [27] hepatocellular carcinoma [28–31], and the high expression of VCAN is positively correlated with the high stage, low differentiation, high metastasis rate and poor prognosis of the tumor. Salem et al. [32] found that low FOXA2/high VCAN levels mediated the pro-proliferation, migration, and invasion effects of mir-590-3P and were negatively related to the survival rate of ovarian cancer. Expression of interstitial VCAN is associated with the accumulation of tumor-related macrophages and the pro-inflammatory and pro-angiogenic state of primary tumors in advanced breast cancer. VCAN can promote the increase in pulmonary metastatic nodules [33]. Jiang et al. [31] used bioinformatics methods to excavate potential prognostic markers of gastric cancer and suggested that VCAN expression was up-regulated in gastric cancer patients with a poor survival time, the same as the results of this study. Our study found that VCAN protein was up-regulated in gastric cancer compared to that in gastritis and positively associated with tumor invasion depth and HER2 protein expression. The relation between VCAN and HER2 protein expression needs further research.

Although we had screened eight different genes between gastric cancer and gastritis, the cause of the changes and their relationships still need further study. Whether THBS2 and VCAN have mutual cooperation in promoting the growth of gastric cancer needs further study too. In summary, THBS2 and VCAN may be potential targets for improving gastric cancer patients' diagnosis and clinical efficacy.
